# Discordance Between Conventional Ultrasound and Transient Elastography in Hepatic Steatosis Assessment: Clinical Factors Associated with Discrepant Findings

**DOI:** 10.3390/diagnostics16081188

**Published:** 2026-04-16

**Authors:** Mihaela Cristina Brisc, Elena Emilia Babeș, Sabina Florina Călugăr-Șolea, Simona Bota, Laura Maghiar, Ciprian Mihai Brisc, Ciprian Brisc

**Affiliations:** 1 Doctoral School of Biomedical Sciences, Faculty of Medicine and Pharmacy, University of Oradea, 410087 Oradea, Romania; brisccristina@uoradea.ro (M.C.B.); eebabes@uoradea.ro (E.E.B.); briscciprian@uoradea.ro (C.B.); 2 Department of Medical Disciplines, Faculty of Medicine and Pharmacy, University of Oradea, 410073 Oradea, Romania; 3 Department of Internal Medicine and Gastroenterology (IMuG), Division of Hepatology, Endocrinology, Rheumatology, Nephrology and Emergency Medicine (ZAE), Centralized Endoscopy Service, Klinikum Klagenfurt am Wörthersee, 9020 Klagenfurt, Austria; bota.simo1982@gmail.com; 4 Department of Psycho-Neurosciences and Rehabilitation, Faculty of Medicine and Pharmacy, University of Oradea, 410073 Oradea, Romania; laura.maghiar@uoradea.ro; 5 Faculty of Medicine and Pharmacy, University of Oradea, 410073 Oradea, Romania

**Keywords:** liver steatosis, liver fibrosis, viral hepatitis, conventional ultrasound, transient elastography, Fib 4 score, serum total cholesterol, metabolic syndrome

## Abstract

**Background**: Discrepancies are frequently observed between liver steatosis grading assessed by conventional B-mode ultrasonography and vibration-controlled transient elastography (VCTE) with controlled attenuation parameter (CAP). This study aimed to identify factors associated with these differences and to evaluate whether the two imaging methods provide comparable steatosis classifications. **Methods**: We conducted a retrospective cross-sectional observational study including 130 hospitalized patients evaluated over a two-year period who underwent laboratory testing, abdominal ultrasonography, and transient elastography. The analyzed variables included demographic characteristics, nutritional status, comorbidities, and biochemical parameters such as alanine aminotransferase (ALAT), total cholesterol, triglycerides, gamma-glutamyl transferase (GGT), and the fibrosis-4 index (FIB-4). Patients were classified into two groups: concordant steatosis grading between the two methods (*n* = 61) and discordant results (*n* = 69). **Results**: Concordant steatosis grading was more frequently observed in patients with serum total cholesterol > 200 mg/dL (45.9%) and FIB-4 values between 1.45–3.25 (44.2%). A trend toward higher concordance was also observed in patients with elevated triglycerides. In contrast, viral liver disease was significantly associated with discordant results (26.2%). Higher fibrosis stages assessed by VCTE (F ≥ 2) and FIB-4 values > 3.25 showed a non-significant trend toward discordance. **Conclusions**: Several clinical and biochemical factors influence the agreement between ultrasound and VCTE-based CAP in the assessment of hepatic steatosis. Elevated cholesterol and intermediate FIB-4 values were associated with concordant results, whereas viral liver disease was associated with discordance between the two imaging modalities.

## 1. Introduction

Fatty liver disease represents a growing concern in modern pathology and is defined as accumulation of lipid molecules in hepatocytes, impairing liver function. The liver plays a central role in systemic lipid metabolism, and disturbances in these pathways may lead to excessive lipid accumulation within hepatocytes, resulting in hepatic steatosis.

From a pathological perspective, fatty liver disease encompasses a spectrum ranging from simple steatosis to steatohepatitis and, in more advanced stages, fibrosis and cirrhosis. Etiologically, hepatic fat accumulation is associated with several conditions, including excessive alcohol intake, hepatitis C virus infection, and metabolic disorders [1]. Diagnostic approaches vary from basic clinical and laboratory evaluation to advanced qualitative and quantitative imaging techniques. Nevertheless, the mechanisms determining why some individuals remain at the stage of simple steatosis while others progress to steatohepatitis or cirrhosis remain incompletely understood.

Histological assessment remains the reference standard for the evaluation of hepatic fat content. In normal liver tissue, fat is present in less than 5% of hepatocytes [1]. Hepatic steatosis is defined by triglyceride accumulation in more than 5% of hepatocytes without hepatocellular ballooning or necrosis [2]. According to the proportion of affected hepatocytes, steatosis is conventionally graded as mild (5–33%), moderate (33–66%), or severe (>66%) [3,4]. When steatosis is accompanied by inflammation and fibrosis, the condition is defined as steatohepatitis [2].

Distinguishing alcoholic from non-alcoholic fatty liver disease may be difficult, especially in former alcohol users. The pathological substrate includes inflammation, necrosis, fibrosis, and features such as Mallory–Denk bodies, ballooning, activated Kupffer cells, acidophil bodies, iron deposition, or lipo-granulomas, spanning alcoholic hepatitis, MASH (Metabolic Dysfunction—Associated Steatohepatitis), viral hepatotropic infections, inherited metabolic disorders, and reactive hepatitis [5]. These overlapping pathological features may contribute to variability in the assessment and grading of hepatic steatosis, highlighting the importance of accurate and reliable diagnostic methods.

The term “liver fat overload” therefore includes both alcoholic and nonalcoholic forms. MASLD (Metabolic Dysfunction—Associated Steatotic Liver Disease) is strongly associated with metabolic syndrome, type 2 diabetes mellitus, and obesity, as widely documented in international studies [6,7,8,9,10,11,12]. Several non-invasive scores (Fatty Liver Index, Hepatic Steatosis Index, SteatoTest, MASLD liver fat score) have been developed for screening purposes, although they cannot precisely quantify hepatic fat.

Imaging techniques used for the evaluation of hepatic steatosis include CT (Computed Tomography), qualitative and quantitative ultrasound methods (Attenuation Imaging, Speed of Sound), and magnetic resonance modalities such as MRI-PDFF (Magnetic Resonance Imaging—Proton Density Fat Fraction) and MRS (Magnetic Resonance Spectroscopy)—considered the most accurate but also costly and less accessible [6]. Liver biopsy remains the gold standard, although it is limited by sampling variability, with documented differences between left and right lobe specimens [5].

In daily clinical practice, the most commonly used rapid tools for diagnosing liver steatosis are B-mode ultrasound and transient elastography, which evaluate fibrosis and the Controlled Attenuation Parameter (CAP), providing information on hepatic fat content [13]. Although some studies have reported suboptimal performance of transient elastography and CAP in assessing steatosis [14], numerous others have demonstrated strong correlations between CAP-derived fat quantification and liver biopsy findings [15,16,17,18,19,20,21,22,23], as well as with Magnetic Resonance Imaging-based assessments [24,25].

However, discrepancies between steatosis grading obtained by conventional ultrasound and elastography-derived CAP may occur in clinical practice. Understanding the factors that may influence these differences is important for improving the diagnostic evaluation of hepatic steatosis.

The aim of this paper is to identify the factors causing concordance and discrepancies between the two steatosis assessment methods and to determine whether their results should align. To achieve this, we conducted a retrospective study evaluating the correlations between conventional B-mode ultrasound and the controlled attenuation parameter measured by transient elastography—as well as their relationships with various clinical and paraclinical parameters.

## 2. Materials and Methods

### 2.1. Study Design and Population

We conducted a cross-sectional, retrospective observational study including 130 patients admitted to the Clinical County Emergency Hospital Bihor, Romania, in the Internal Medicine and Gastroenterology Departments between February 2023 and February 2025.

Inclusion criteria: All included participants underwent same-day laboratory testing, abdominal B-mode ultrasound, and transient elastography using the extra-large probe (XL). Ultrasound and FibroScan (Echosens, Paris, France) examinations were performed in fasting patients, in the morning of admission, after blood sampling and prior to the administration of chronic medication.

Exclusion criteria: Patients were excluded if the two imaging methods were not performed on the same day, if the M probe was required, or if active oncological disease was present.

The baseline characteristics of the included patients are presented in Table 1. Data are presented as number (percentage).

### 2.2. Ultrasound Examination

Liver ultrasound (US) evaluation was performed according to international standards, with patients examined in the supine position using sagittal, transverse, and oblique sections on a Hitachi (Hitachi, Tokyo, Japan) system equipped with a 1–5 MHz convex probe. All examinations were conducted by the same operator, who assessed liver size, surface, echogenicity, echotexture, hepatic and portal vein caliber, and the presence of normal hepato-petal portal flow.

The operator performing the ultrasound examination was not aware of the CAP-derived steatosis grading at the time of the ultrasound assessment, because US assessment was performed before Transient Elastography was. The present sequence of paraclinical investigations was also selected in order to identify potential anatomical variations in hepatic positioning, thereby minimizing the risk of obtaining invalid Fibro Scan measurements.

Steatosis grading followed established ultrasonographic criteria, including increased liver brightness, reduced visualization of hepatic veins, focal fat sparing, and diminished visualization of the diaphragm and deep parenchyma [26,27,28,29,30,31]. Mild steatosis (grade 1) was defined by increased echogenicity; moderate steatosis (grade 2) by posterior beam attenuation; and severe steatosis (grade 3) by poor visualization of deep hepatic veins, diaphragm, or deep parenchyma [30,31].

### 2.3. Transient Elastography and CAP Measurement

Transient elastography was performed after ultrasound, using standard protocols, with patients in the supine position and measurements obtained in the 9th–10th intercostal space along the mid-axillary line. Ultrasound preceded elastography to ensure optimal probe placement over the right hepatic lobe.

Assessments were conducted using the Echosens FibroScan (XL probe), which provides liver stiffness and steatosis estimation via the Controlled Attenuation Parameter (CAP). Only measurements with ≥10 valid readings, a success rate > 60%, and an interquartile range < 30% of the median were included. All transient elastography examinations included in the study were performed using the XL probe. This approach was adopted in order to ensure methodological consistency and to minimize potential variability related to probe selection. In some patients with lower BMI, the XL probe was used when adequate intercostal acoustic windows were available, allowing reliable measurements while maintaining a uniform acquisition protocol across the study population.

Steatosis and fibrosis were classified using the Interpretation Guide developed by Echosens, which is based on internationally accepted cut-off values for the Controlled Attenuation Parameter (CAP) and liver stiffness measurement (LSM), as validated across different liver disease etiologies, including metabolic dysfunction–associated steatosis liver disease (MASLD), metabolic dysfunction–associated steatohepatitis (MASH), viral hepatitis, alcohol-related liver disease, autoimmune hepatitis, and cholestatic liver diseases. Steatosis was assessed using CAP values (expressed in dB/m) and graded as follows: S0 < 248 dB/m, S1 248–267 dB/m, S2 268–279 dB/m, and S3 ≥ 280 dB/m. Fibrosis staging was based on LSM values (expressed in kPa) and defined as: F0–F1 < 7.0 kPa, F2 7.0–9.5 kPa, F3 9.5–12.5 kPa, and F4 ≥ 12.5 kPa.

### 2.4. Definition of Concordance and Discordance

Concordance between conventional ultrasound and transient elastography–derived CAP measurements was defined as the assignment of the same steatosis grade by both imaging modalities.

Discordance was defined as any difference in steatosis grading between the two techniques. Given the ordinal nature of steatosis grading, the extent of discordance was further characterized by recording the number of grading categories separating the two methods, distinguishing between a one-grade difference and discrepancies of two or more grades.

We acknowledge that a one-grade difference may partly reflect the inherent variability of ultrasound-based assessment; however, this definition was adopted to capture the full spectrum of potential discrepancies between the two imaging techniques. In addition, an exploratory analysis using a stricter definition of discordance (≥2 grades) was performed.

Based on these criteria, patients were categorized into two groups: Group 1, including cases with concordant steatosis grading, and Group 2, including cases with discordant steatosis grading between the two imaging modalities.

### 2.5. Clinical and Biochemical Variables

Collected variables included age, sex, residential environment, nutritional status, hepatic, metabolic, and cardiovascular comorbidities, as well as some biochemical markers (ALAT, GGT, total cholesterol, triglycerides). Viral etiology included patients with chronic hepatitis B or chronic hepatitis C infection, diagnosed based on standard serological criteria. The patients with C virus infection were patients with finalized treatment and with obtained SVR. All patients with B virus infection were receiving ongoing antiviral therapy with nucleos(t)ide analogues.

Fibrosis severity was estimated using the FIB-4 score as follows: FIB-4 = age (years) × ASAT (U/L)/Platelets (109 /L) × √ALAT (U/L) [26]. Laboratory investigations were performed using the automated Abbott diagnostic platform (Abbott Laboratories, Abbott Park, IL, USA). All clinical assessments, laboratory analyses, and imaging procedures were carried out on the same day, using the same equipment and operator, to reduce procedural variability.

### 2.6. Statistical Analysis

Statistical analysis was performed using MedCalc Statistical Software 23.5.2 (MedCalc Software Ltd., Ostend, Belgium). The distribution of numerical variables was assessed using the Kolmogorov–Smirnov test. Continuous variables were expressed as mean ± standard deviation or median (range), as appropriate. Comparisons between groups were performed using Student’s t-test, Mann–Whitney test, or Kruskal–Wallis test, as appropriate. Categorical variables were compared using the chi-square test or Fisher’s exact test. A *p* value < 0.05 was considered statistically significant.

Factors associated with discordance between liver steatosis assessed by abdominal ultrasound and transient elastography were evaluated by univariate analysis. Variables with *p* < 0.1 were included in a multivariable logistic regression model to identify independent predictors of concordant steatosis grading. Results were expressed as odds ratios (ORs) with 95% confidence intervals (CIs).

Agreement between ultrasound and CAP-derived steatosis grading was assessed using a 4 × 4 contingency table and quantified with weighted kappa statistics (quadratic weights).

For ordinal variables with more than two categories, comparisons between concordant and discordant groups were performed using overall tests across categories.

In the multivariable model, FIB-4 was included as a continuous variable.

## 3. Results

A total of 130 patients were included in the study. The baseline characteristics of the study population are presented in Table 1.

**Figure 1:**
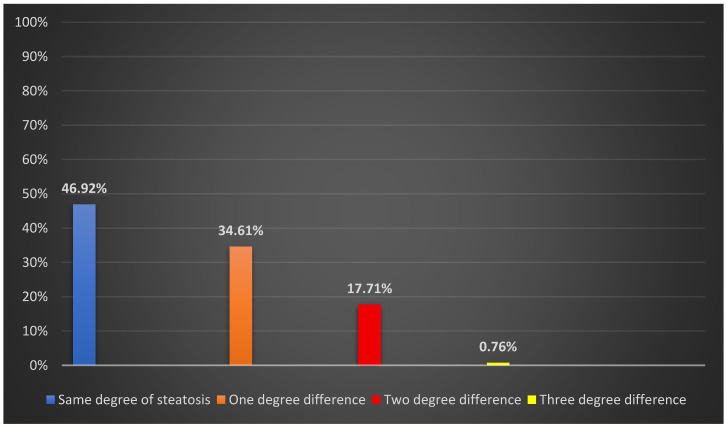
Distribution of concordant and discordant steatosis grading between ultrasound and CAP.

Of the 130 patients, 61 (46.92%) showed concordant steatosis grading between the two non-invasive methods (ultrasound and transient elastography), whereas 69 patients (53.08%) had discordant results. Among the discordant cases, 45 patients (34.61% of the total cohort) exhibited a one-grade discrepancy, 23 patients (17.71%) had a two-grade discrepancy, and 1 patient (0.76%) had a three-grade discrepancy (Figure 1).

Discordance is further stratified according to the magnitude of difference.

Among the discordant cases (*n* = 69), ultrasound yielded a higher steatosis grade than CAP in 48 patients (69.56%), whereas CAP provided a higher grade than ultrasound in 21 patients (30.44%).

This finding indicates that most discordant cases involved only a one-grade difference, whereas larger discrepancies were less frequent. Discordance was also analyzed using a stricter definition (≥2 grades difference). Under this definition, 24 patients (18.47%) were classified as having major discordance between ultrasound and CAP. Due to the limited number of cases with ≥2-grade differences and the original study design, no formal univariate or multivariate analyses were performed. The findings related to this subgroup should therefore be interpreted as exploratory.

To assess the level of agreement between the two imaging modalities, the full distribution of steatosis grades obtained by conventional ultrasound and CAP was analyzed using a 4 × 4 contingency table (Table 2). Agreement analysis based on this distribution of steatosis grades demonstrated substantial concordance between the two techniques (weighted κ = 0.62), according to the Landis and Koch criteria.

To explore the factors associated with this variability, the study population was divided into two groups: Group 1, including patients with concordant steatosis grading between conventional ultrasound and transient elastography (*n* = 61), and Group 2, including patients with discordant results (*n* = 69).

For ordinal variables such as age categories, BMI, fibrosis stage, and FIB-4 score, statistical comparisons between groups were performed using global tests rather than category-specific analyses, in order to account for the ordinal structure of the data and avoid multiple comparisons.

Gender, environment, age, alcoholic and autoimmune etiology, BMI, cardio-metabolic comorbidities (such as heart failure, ischemic heart disease, atrial fibrillation, hypertension, ectopic heart beats, dilated cardiomyopathy), the level of ALAT and fibrosis risk by estimated by FIB-4 score (<1.45 or >3.25), were not significantly associated with concordance or discordance between the two imaging methods (Table 3).

In the univariate analysis, elevated total serum cholesterol (>200 mg/dL) was significantly associated with concordant steatosis grading (*p* = 0.02). A trend toward higher concordance was also observed in patients with elevated serum triglycerides (>150 mg/dL), although this did not reach statistical significance (*p* = 0.07) (Table 3).

Conversely, viral etiology was significantly associated with discordant steatosis grading (*p* = 0.01). Fibrosis-related variables, including elastography-defined fibrosis stage ≥ F2 and higher FIB-4 categories, showed a trend toward discordance; however, these associations did not reach statistical significance (*p* = 0.055 and *p* = 0.09, respectively) (Table 3). This subgroup analysis is exploratory in nature and should be interpreted with caution due to the limited sample size.

Multivariable logistic regression analysis identified serum cholesterol levels > 200 mg/dL as an independent predictor of concordant steatosis grading (OR 2.23, 95% CI 1.07–4.66, *p* = 0.03), whereas viral etiology and FIB-4 were not significant predictors (Table 4).

## 4. Discussions

The present study evaluated the agreement between conventional ultrasound and transient elastography-derived CAP in the assessment of hepatic steatosis in a real-world clinical setting.

Overall, concordant steatosis grading between the two imaging modalities was observed in 61 of 130 patients (46.92%), whereas discordant results occurred in 69 patients (53.08%).

Agreement analysis based on the full distribution of steatosis grades demonstrated substantial concordance between the two techniques (weighted κ = 0.62), according to the Landis and Koch classification. Notably, most discordant cases involved only a one-grade difference, suggesting that these discrepancies are generally minor and may partly reflect the semi-quantitative nature of ultrasound-based steatosis grading.

Among discordant cases, ultrasound yielded a higher steatosis grade than CAP in the majority of patients (69.56%). This directional difference may be explained by the fundamental differences in sampling between the two methods. Conventional ultrasound provides a qualitative assessment of steatosis across the entire liver parenchyma, whereas CAP measurements reflect a more localized region of interest. This may lead to discrepancies, particularly in the presence of heterogeneous fat distribution or structural liver alterations, such as fibrosis or inflammation.

Several variables did not show a statistically significant association with concordance or discordance between the two imaging methods, including age, sex, environment, alcoholic and autoimmune etiology, and mild fibrosis assessed by transient elastography.

In line with previous studies, body mass index (BMI) [32], the presence of type 2 diabetes mellitus, arterial hypertension, and other cardiovascular or metabolic comorbidities [33], such as hypothyroidism, were not associated with differences in steatosis grading. In addition, contrary to some reports [34,35], serum aminotransferase levels did not appear to be a reliable indicator of discordance between ultrasound and transient elastography in the assessment of hepatic steatosis which is consistent with the findings of another study reporting similar results [36].

Our Results showed several clinical and biochemical factors which were associated with agreement or discordance between the two imaging modalities, including viral etiology, elevated serum cholesterol levels, and FIB-4 values.

In univariate analysis, viral etiology was significantly associated with discordant steatosis grading. However, this association was not confirmed in multivariate analysis and should therefore be interpreted with caution. This finding may reflect the complex interplay between inflammation, fibrosis, and tissue heterogeneity in viral hepatitis, which can influence imaging-based steatosis assessment [37,38,39,40,41,42,43,44].

Another important aspect is the variability of CAP diagnostic thresholds. Although the cutoffs used in the present study are widely reported, optimal thresholds may vary depending on disease etiology, BMI, and fibrosis stage. In particular, CAP performance may differ in patients with chronic viral hepatitis or advanced fibrosis, suggesting that fixed cutoff values may not be uniformly applicable across clinical contexts [42,43,44].

Elevated serum cholesterol levels (>200 mg/dL) were significantly associated with concordant steatosis grading. Although hypercholesterolemia is not a direct marker of hepatic fat accumulation, it may reflect underlying metabolic alterations and should be interpreted with caution.

High triglyceride levels (>150 mg/dL) showed a non-significant trend toward greater concordance, suggesting that a metabolically driven lipid profile may favor more consistent steatosis assessment. These findings underscore the role of metabolic factors in the interpretation of non-invasive imaging results [45,46,47]. Elevated triglycerides are associated with diffuse steatosis [48,49], which may result in more uniform hepatic echogenicity and attenuation.

Fibrosis stage, as estimated by the FIB-4 score, showed a differential relationship with concordance. Intermediate values were associated with discordance, whereas higher values showed only a non-significant trend as patients with discordant steatosis grading tended to present a higher prevalence of advanced fibrosis. Similar variability between non-invasive fibrosis assessment tools has been reported in recent studies [50].

Fibrosis-related architectural changes, including collagen deposition, heterogeneous fat distribution, and altered acoustic properties, may contribute to discrepancies between imaging modalities [51,52,53,54]. However, our fibrosis-related variables did not retain statistical significance in multivariate analysis, possibly due to collinearity with other markers of liver disease severity, and its role should be interpreted cautiously.

Overall, concordance between ultrasound and CAP-based steatosis assessment appears more likely in patients with a metabolically driven lipid profile, whereas discordance tends to occur in the context of viral liver disease and increasing fibrosis severity. This pattern may reflect differences in hepatic fat distribution, which is typically diffuse in metabolic steatosis but more heterogeneous in the presence of inflammation and fibrosis, where structural alterations of the hepatic microarchitecture may differentially influence imaging measurements.

These results indicate that clinical and biochemical factors may influence agreement between non-invasive imaging techniques. Further prospective studies incorporating reference standards such as MRI-PDFF [55] or liver biopsy, as well as larger and more homogeneous cohorts, are needed to better understand the mechanisms underlying these discrepancies.

## 5. Limitation of the Study

This study has several limitations. The absence of a reference standard (MRI-PDFF or liver biopsy) precludes assessment of the relative accuracy of the two imaging methods, and the analysis therefore focuses on discordance and associated factors. Only examinations performed with the XL probe were included, which may limit generalizability but ensured methodological consistency. In addition, all ultrasound examinations were performed by a single experienced operator, precluding assessment of interobserver variability. The viral hepatitis subgroup was heterogeneous and lacked detailed characterization of inflammatory activity, which may influence imaging measurements. Finally, the inclusion of hospitalized patients may introduce bias, as acute illness or systemic inflammation could affect liver parameters.

## 6. Conclusions

Concordance between conventional ultrasound and transient elastography in the assessment of hepatic steatosis appears to be influenced by underlying clinical and metabolic factors. Elevated serum cholesterol levels were independently associated with concordant steatosis grading, whereas other variables, including triglyceride levels and fibrosis-related parameters, demonstrated only non-significant trends.

Discordance between the two methods was more frequently observed in the context of viral liver disease and increasing fibrosis burden; however, these associations were not retained in multivariate analysis and should therefore be interpreted with caution.

Collectively, these findings suggest that the underlying liver disease phenotype, particularly metabolic versus non-metabolic patterns, may influence the level of agreement between non-invasive imaging techniques for steatosis assessment.

## Figures and Tables

**Table 1:** Baseline characteristics of the study population.

Parameter	Total of Patients *n* = 130
Gender Male gender Female gender	62 (47.69%) 68 (52.31%)
Urban area Rural area	72 (55.38%) 58 (44.62%)
Age (years) < 50 50–69 ≥70	30 (23.07%) 72 (55.38%) 28 (21.55%)
Etiology viral alcoholic autoimmune other	24 (18.46%) 18 (13.84%) 2 (1.53%) 86 (66.17%)
Fibrosis (measured by TE) F0 F1 F2 F3 F4	39 (30.00%) 62 (47.69%) 9 (6.92%) 6 (4.61%) 14 (10.78%)
BMI (kg/m^2^) <20 20–24.9 25–29.9 ≥30	6 (4.61%) 24 (18.46%) 52 (40.00%) 48 (36.93%)
Type 2 Diabetes mellitus	43 (33.07%)
Cardiac comorbidities	21 (16.15%)
Arterial Hypertension	72 (55.38%)
Hypothyroidism	16 (12.30%)
ALAT > ULN	23 (17.69%)
GGT > ULN	32 (24.61%)
Total Serum Cholesterol > 200 mg/dL	47 (36.15%)
Triglycerides > 150 mg/dL	37 (28.46%)
FIB-4 < 1.45	69 (53.07%)
1.45–3.25	46 (35.38%)
>3.25	15 (11.50%)

TE—transient elastography; F—fibrosis stage; BMI—body mass index; ALAT—alanine aminotransferase; ULN—Upper Limit of Normal; GGT—gamma-glutamyl transferase; FIB 4—Fibrosis 4 index.

**Table 2:** Cross-tabulation of steatosis grades between ultrasound and CAP.

	CAP S0	CAP S1	CAP S2	CAP S3	Total
US S0	27	3	1	1	32
US S1	12	3	4	3	22
US S2	19	13	6	10	48
US S3	0	0	3	25	28
Total	58	19	14	39	130

CAP: Controlled Attenuation Parameter; S: Steatosis grade.

**Table 3:** Univariate analysis of factors associated with concordant steatosis grading.

Variable	Concordance US-TE (*n* = 61) Group 1	Discordant US-TE (*n* = 69) Group 2	*p* Value
Male gender	33 (54.1%)	29 (42.1%)	0.17
Urban area	33 (54.1%)	39 (56.5%)	0.78
Age (years)			Global *p* = 0.41
<50	16 (26.2%)	14 (20.3%)	0.42
50–69	30 (49.2%)	42 (60.8%)	0.18
≥70	15 (24.6%)	13 (18.9%)	0.43
Etiology			Global *p* = 0.54
viral	6 (9.8%)	18 (26.1%)	0.01
alcoholic	7 (11.4%)	11 (15.9%)	0.45
autoimmune	1 (1.6%)	1 (1.5%)	0.96
Fibrosis (TE)			Global *p* = 0.38
F0	20 (32.8%)	19 (27.6%)	0.52
F1	32 (52.4%)	30 (43.4%)	0.30
F2	2 (3.3%)	7 (10.1%)	0.12
F3	2 (3.3%)	4 (5.8%)	0.50
F4	5 (8.2%)	9 (13.1%)	0.37
Fibrosis (TE) ≥ 2	9 (14.8%)	20 (29%)	0.055
BMI (kg/m^2^)			Global *p* = 0.36
<20	4 (6.6%)	2 (2.9%)	0.31
20–24.9	8 (13.1%)	16 (23.2%)	0.14
25–29.9	24 (39.4%)	28 (40.6%)	0.88
≥30	25 (40.9%)	23 (33.3%)	0.37
Type 2 Diabetes mellitus	22 (36.1%)	22 (30.4%)	0.49
Cardiac comorbidities	8 (13.1%)	13 (18.8%)	0.43
Arterial Hypertension	34 (55.7%)	38 (55.1%)	0.94
ALAT > ULN	10 (16.4%)	13 (18.8%)	0.72
GGT > ULN	13 (21.3%)	19 (27.5%)	0.41
Total Serum Cholesterol > 200 mg/dL	28 (45.9%)	19 (27.5%)	0.02
Serum Triglycerides > 150 mg/dL	22 (36.1%)	15 (21.7%)	0.07
FIB-4			Global *p* = 0.069
<1.45	30 (49.2%)	39 (56.6%)	0.40
1.45–3.25	27 (44.2%)	19 (27.5%)	0.04
>3.25	4 (6.6%)	11 (15.9%)	0.09

TE—transient elastography; F—fibrosis stage; BMI—body mass index; ALAT—alanine aminotransferase; ULN—Upper Limit of Normal; GGT—gamma-glutamyl transferase; FIB-4—fibrosis 4 index. Data are presented as *n* (%). For ordinal variables with more than two categories, global *p* values were calculated using overall comparisons across categories.

**Table 4:** Multivariable logistic regression analysis of factors associated with concordant steatosis grading between ultrasound and transient elastography.

Variable	Odds Ratio (OR)	95% CI	*p* Value
Viral etiology	0.59	0.18–1.95	0.37
Cholesterol > 200 mg/dL	2.23	1.07–4.66	0.03
FIB-4	0.98	0.59–1.63	0.92

## Data Availability

Patients’ information is private and is archived in the electronic databases of the medical units where the research was done.

## Abbreviations

The following abbreviations are used in this manuscript:

ALAT	Alanine Aminotransferase
ASAT	Aspartate Aminotransferase
BMI	Body Mass Index
CAP	Controlled Attenuation Parameter
CI	Confidence interval
CT	Computed Tomography
F0	Stage 0 of Liver Fibrosis or No Liver Fibrosis
F1	Stage 1 of Liver Fibrosis
F2	Stage 2 of Liver Fibrosis
F3	Stage 3 of Liver Fibrosis
F4	Stage 4 of Liver Fibrosis, or Advance Liver Fibrosis, or Liver Cirrhosis
FIB 4 score	Fibrosis 4 Index
GGT	Gamma-Glutamyl Transferase
LSM	Liver Stiffness Measurement
MASH	Metabolic Dysfunction-Associated Steatohepatitis
MASLD	Metabolic Dysfunction-Associated Steatotic Liver Disease
MRI	Magnetic Resonance Imaging
MRI-PDFF	Magnetic Resonance Imaging-Proton Density Fat Fraction
MRS	Magnetic Resonance Spectroscopy
OR	Odds ratio
TE	Transient elastography
T2DM	Type 2 Diabetes Mellitus
US	Ultrasound
VCTE	Vibration-Controlled Transient Elastography
XL probe	Extra-Large probe
WK	Weighted Kappa
